# New Publications

**Published:** 1902-02

**Authors:** 


### NEW PUBLICATIONS.

*Bulletin No. 79* of the State Live-stock Sanitary Board adds more literature to the subject of "rabies." It is prepared by the bacteriologist of the Board, Dr. Mazyck P. Ravenel. It covers the history, distribution, animals affected, causes, treatment, prevention, and eradication. If the English-speaking veterinarians of the world were supplied with literature on all other subjects as prolific as that which is extant to-day on rabies and hydrophobia one's library would reach very large dimensions.

### NOTES.

There is pending in the Legislature of Massachusetts a bill to abolish the Board of Cattle Commissioners and to substitute for it a Cattle Bureau, with a chief in the Board of Agriculture. It is thus proposed that all of the work of the commission shall be vested in one man.

This move is in harmony with the present spirit of concentration and is practically what a great many States have done in appointing State Veterinarians. Multiheaded executive boards are often unsatisfactory.

There is in the law an omission that we regard as serious. It is not provided that the chief of the cattle bureau shall be a veterinarian, although all of his duties will be in the administration of veterinary work. Of course, it is probable that the Governor will appoint a veterinarian to this position, but it would have been better had this requirement been placed in the bill.

The present commission which is to be abolished is composed of two farmers, one of these a cow-dealer and one a veterinarian.

It appears probable at this writing that the bill will pass, for it is recommended by the Governor in his message and is reported to the Senate jointly by the Committees on Agriculture and Public Service.

A bill has been introduced in the Massachusetts Legislature to abolish the Board of Cattle Commissioners and establish a Bureau of Cattle, with a chief as its head, in place of the three Commissioners. The proposition has the approval of the Governor. What is the attitude of the Bay State veterinarians in the matter?

The new organization of veterinarians of Cuyahoga County, Ohio, are actively instituting actions against a number of illegal practitioners of veterinary medicine, under the laws of Ohio regulating the practice of the veterinary profession.

The Pennsylvania State Board of Veterinary Medical Examiners instituted several suits against alleged illegal practitioners in York and Lancaster counties in February. The successful prosecutions in Schuylkill and Bedford counties has had a very salutary effect.

Columbus, Nebraska, reports the death of a large number of horses from some unusual symptoms—sudden blindness, loss of nervous power, animals running into barbed-wire fences, loss of sensation, cold sweats, and death in a few hours.

